# A *p*-Adic Model of Quantum States and the *p*-Adic Qubit

**DOI:** 10.3390/e25010086

**Published:** 2022-12-31

**Authors:** Paolo Aniello, Stefano Mancini, Vincenzo Parisi

**Affiliations:** 1 Dipartimento di Fisica “Ettore Pancini”, Università di Napoli “Federico II”, Complesso Universitario di Monte S. Angelo, Via Cintia, I-80126 Napoli, Italy; 2 Istituto Nazionale di Fisica Nucleare, Sezione di Napoli, Complesso Universitario di Monte S. Angelo, Via Cintia, I-80126 Napoli, Italy; 3 School of Science and Technology, University of Camerino, Via Madonna delle Carceri, 9, I-62032 Camerino, Italy; 4 Istituto Nazionale di Fisica Nucleare, Sezione di Perugia, Via A. Pascoli, I-06123 Perugia, Italy

**Keywords:** ultrametric field, *p*-adic quantum mechanics, quantum state, *p*-adic probability

## Abstract

We propose a model of a quantum N-dimensional system (quNit) based on a quadratic extension of the non-Archimedean field of *p*-adic numbers. As in the standard complex setting, states and observables of a *p*-adic quantum system are implemented by suitable linear operators in a *p*-adic Hilbert space. In particular, owing to the distinguishing features of *p*-adic probability theory, the states of an N-dimensional *p*-adic quantum system are implemented by *p*-adic statistical operators, i.e., trace-one selfadjoint operators in the carrier Hilbert space. Accordingly, we introduce the notion of selfadjoint-operator-valued measure (SOVM)—a suitable *p*-adic counterpart of a POVM in a complex Hilbert space—as a convenient mathematical tool describing the physical observables of a *p*-adic quantum system. Eventually, we focus on the special case where N=2, thus providing a description of *p*-adic qubit states and 2-dimensional SOVMs. The analogies—but also the non-trivial differences—with respect to the qubit states of standard quantum mechanics are then analyzed.

## 1. Introduction

The field of *p*-adic numbers $Q_{p}$ was introduced by K. Hensel at the end of the XIX century, mainly in connection with pure mathematical problems. The peculiarity of this field, in sharp contrast with the fields of real and complex numbers R and C, is its natural *ultrametric structure*, that entails a non-Archimedean character of this field. It came as a complete surprise when, at the end of the past century, some concrete applications of *p*-adic numbers to physical theories began to appear. Indeed, in the late 1980s, Vladimirov, Volovich and Zelenov [1,2] argued that the existence of a smallest measurable length—i.e., the so-called Planck length $l_{P}\approx 10^{-35}$ m, predicted in quantum gravity and string theory, see [3] and references therein—forces one to adopt a *non-Riemannian model*, that is, a model in which the Archimedean property is no more valid at very small distances. In particular, they proposed a model of quantum mechanics based on the non-Archimedean field of *p*-adic numbers. Later on, different *p*-adic quantum mechanical models were studied [4,5,6,7,8,9,10,11,12], and several applications to quantum field theory were proposed [13,14,15,16,17,18]. More or less in the same years, other unexpected connections between *p*-adic numbers and theoretical physics were revealed. e.g., it was argued that the natural *fractal-like* structure of this field makes it suitable for the description of the dynamics of chaotic and disordered systems. In particular, it was proved that the ground state of *spin glasses* exhibits a natural (non-Archimedean) ultrametric structure [19,20,21].

More recently, new and interesting applications of *p*-adic numbers, not necessarily related to foundational physics, have begun to appear. Indeed, *p*-adic numbers have found a fertile ground of application in the context of *algebraic dynamical systems*, also in connection with problems from computer science, image analysis, compression of information, image recognition and cryptography [22,23,24,25]. In particular, one of the most prominent applications to computer science and cryptography is related to the generation of *pseudorandom numbers* and *uniform distribution of sequences* [25,26,27,28].

A very recent research trend involves applications to quantum information theory [29], as well. The interest for a formulation of a *p*-adic quantum information theory is two-fold: On the one hand, the peculiarities of *p*-adic numbers may provide a new line of attack for notoriously hard problems in quantum information theory; e.g., it has been recently argued that *p*-adic numbers (or, more generally, *m*-adic numbers) can be profitably used in the construction of mutually unbiased bases (MUBs), in any given Hilbert space dimension [30]. On the other hand, a *p*-adic model of quantum information may provide useful tools for the study of fundamental physical theories.

Until today, it seems, however, that no general model of quantum information based on *p*-adic numbers has been formulated. We believe that the main reason for this is the lack of a well established theory of quantum states in the *p*-adic setting. Recently, this issue has been addressed in [31], where it is demonstrated how the usual density operators of complex quantum mechanics should be replaced, in the *p*-adic framework, by suitable *p*-adic (trace class) operators in a *p*-adic Hilbert space. In the present contribution, on the base of the ideas presented in the aforementioned reference, we propose a *p*-adic model of a quNit. In particular, in these finite dimensional systems, we will show that the set of *p*-adic linear operators has itself a structure of a *p*-adic Hilbert space. This turns out to be useful for describing observables and the measurement process. The case of *p*-adic qubit will be worked out explicitly.

The structure of the paper is as follows. In Section 2, we review some basic notions concerning the field of *p*-adic numbers and its quadratic extensions. In Section 3, we introduce the *p*-adic Hilbert spaces and the associated linear operators. We devote Section 4 to the characterization of *p*-adic states in a finite-dimensional quantum system. Finally, by focusing on the case of a two-dimensional *p*-adic Hilbert space, we obtain an explicit realization of a *p*-adic qubit. Section 5 is for concluding remarks.

## 2. Overview on *p*-Adic Numbers

In this section, we remind the reader some basic notions and results concerning *p*-adic numbers that are relevant for *p*-adic quantum mechanics [1,2,4,5,6,7,9,15]. We also introduce our main notations and terminology.

By a *valuation* (or *absolute value*) on Q we mean a map, |·|:Q→R, such that, for all x,y∈Q,

(V1)
|x|≥0, and |x|=0 iff x=0 (positive definiteness);
(V2)
|xy|=|x||y| (multiplicativity);
(V3)
|x+y|≤|x|+|y| (triangle inequality).

In particular, in the case where |·| verifies the additional condition (actually, a `stronger version’ of (V3))

(V4)
|x+y|≤max{|x|,|y|} (strong triangle inequality),

we say that |·| is a *non-Archimedean valuation*; otherwise, the valuation is called *Archimedean*.

An Archimedean valuation |·| on Q induces a *distance function* (or *metric*) defined by:

(1) $$d_{\left|\cdot \right|}\left(x,y\right)≔\left|x-y\right|.$$

Note that, in the case where in expression (1) the valuation |·| is *non*-Archimedean, instead, we still have a metric on Q, but, the strong triangle inequality (cf. (V4)) entails that the distance $d_{\left|\cdot \right|}$ verifies a stronger condition, namely,

(2) $$d_{\left|\cdot \right|}\left(x,y\right)\leq \max\left\{d_{\left|\cdot \right|}\left(x,z\right),d_{\left|\cdot \right|}\left(z,y\right)\right\},\;\forall x,y,z\in Q.$$

In the mathematical literature, property (2) is usually referred to as *ultrametricity* and, accordingly, a metric function satisfying it is called an *ultrametric*.

**Example 1**  ([1,32,33,34]). *Recall that, according to the unique factorization theorem, every rational number x∈Q can be expressed as $x=p^{k}q$, where p∈N is a fixed prime number, k some integer in Z, and q a rational number whose numerator and denominator are not divisible by p [32,35]. The p-adic absolute value is then defined as the map ${\left|\cdot \right|}_{p}:Q\to R$, such that ${\left|0\right|}_{p}\equiv 0$, and*

(3) $${\left|x\right|}_{p}≔p^{-k},\;\forall x\neq 0.$$

It is easily shown that ${\left|\cdot \right|}_{p}$ is a non-Archimedean valuation on Q, since it is strictly positive on $Q^{*}\equiv Q\backslash \left\{0\right\}$, it factorizes under the product of two elements in Q, and verifies the strong triangle inequality *(V4)*. Therefore, if we consider the associated metric function

(4) $$d_{{\left|\cdot \right|}_{p}}\left(x,y\right)≔{\left|x-y\right|}_{p},$$

*we obtain an ultrametric on Q.*

Consider the pair $\left(Q,d_{{\left|\cdot \right|}_{p}}\right)$, where $d_{{\left|\cdot \right|}_{p}}$ is the ultrametric associated with the *p*-adic valuation (see Example 1). It is a metric space that, by means of a standard procedure, can be completed [36]. The resulting complete field is usually called the field of *p-adic numbers* $Q_{p}$. This is a standard (through rather abstract) way to define *p*-adic numbers. A more concrete characterization is given as follows. Let $x\in Q_{p}^{*}\equiv Q_{p}\backslash \left\{0\right\}$. It is possible to prove that *x* admits a unique decomposition of the form

(5) $$x=\sum_{i=0}^{\infty }x_{i}p^{i+k},\;k\in Z,\;x_{i}\in \left\{0,1,\ldots ,p-1\right\},\;x_{0}\neq 0,$$

and, conversely, every series of this form converges to some non-zero element of $Q_{p}$ [32]. Therefore, we see that the decomposition (5) provides a representation of any *p*-adic number by means of a suitable converging series. In particular, this is reminiscent, to some extent, of the usual decimal expansion of a real number x∈R, namely,

(6) $$x=\pm 10^{k}\left(x_{0}+x_{1}10^{-1}+x_{2}10^{-2}+\ldots \right),\;k\in Z,\;\;x_{i}=0,1,\ldots ,9,\;\;x_{0}\neq 0.$$

The *p*-adic valuation on Q can be extended—in a unique way—to a non-Archimedean valuation on $Q_{p}$ which, still using the same symbol (with a slight abuse of notation), is given by

(7) $${\left|x\right|}_{p}=|\sum_{i=0}^{\infty }x_{i}\;p^{i+k}{|}_{p}=p^{-k},\;\forall x\in Q_{p}^{*}.$$

Clearly, by a similar reasoning, also the ultrametric (4) can be extended to an ultrametric on $Q_{p}$. The ultrametricity condition satisfied by this ultrametric reflects in some topological peculiarities of $Q_{p}$ that, ultimately, justify the use of *p*-adic numbers when describing physics on length scales comparable to Planck’s length $l_{P}$ [1,2,4,5,10,11]. Just to mention the most relevant ones [37,38], we list the following points:

(P1) *Every point in an open (closed) ball is a centre*.
(P2) *Two open (closed) balls are either disjoint or one is contained in the other*.
(P3) *Every ball in $Q_{p}$ is both closed and open (in short, clopen) in the ultrametric topology of $Q_{p}$.*
(P4) *All triangles are isosceles in $Q_{p}$*.

As a topological space, $Q_{p}$ is *completely regular* (being a metric space) and *totally disconnected*; namely, the only connected subsets of $Q_{p}$ are the singletons [37].

We devote the last part of this section to a brief discussion of the *quadratic extensions* of $Q_{p}$. The opportunity of switching to a quadratic extension is related to the lack of a non-trivial involution on $Q_{p}$ [25,33]. This is analogous to the formulation of standard quantum mechanics relying on the field C of complex numbers, with C regarded as a quadratic extension of the reals, and endowed with its natural involution (the complex conjugation).

The definition of a quadratic extension of $Q_{p}$ closely mimics the one given for the field of complex numbers C. Indeed, let $\mu \in Q_{p}$ be a non-quadratic element in $Q_{p}$, i.e., $\mu \notin {\left(Q_{p}^{*}\right)}^{2}$. Introducing the symbol $\sqrt{\mu }$ (which plays a role analogous to the one played by the *imaginary unit* in C), the quadratic extension $Q_{p,\mu }$ of $Q_{p}$ induced by μ is defined as the set

(8) $$Q_{p,\mu }≔\left\{x+\sqrt{\mu }y|x,y\in Q_{p}\right\}.$$

It is easily verified that $Q_{p,\mu }$ is a field extension of $Q_{p}$. Indeed, $Q_{p,\mu }$ is a two-dimensional vector space on $Q_{p}$, its elements can be added and multiplied following the usual rules, and any non-null element admits a unique inverse, which is given by

(9) $${\left(x+y\sqrt{\mu }\right)}^{-1}=\frac{x}{x^{2}-\mu y^{2}}-\sqrt{\mu }\frac{y}{x^{2}-\mu y^{2}},$$

where the denominator $x^{2}-\mu y^{2}$ is not zero (otherwise μ should be a square in $Q_{p}$). On the field $Q_{p,\mu }$, it is possible to define a *conjugation*, namely, the mapping

(10) $$z=x+\sqrt{\mu }y\mapsto \bar{z}≔x-\sqrt{\mu }y,$$

so that

(11) $$z\bar{z}=x^{2}-\mu y^{2}\in Q_{p}.$$

Moreover, the *p*-adic absolute value ${\left|\cdot \right|}_{p}$ can be extended—in a unique way—to a non-Archimedean valuation ${\left|\cdot \right|}_{p,\mu }$ on $Q_{p,\mu }$, which is given by

(12) $${\left|z\right|}_{p,\mu }=\sqrt{|z\bar{z}{|}_{p}}.$$

For the sake of conciseness, henceforth we will simply denote this valuation by |·|. However, differently from the real case, there exist various inequivalent quadratic extensions of $Q_{p}$. In fact, we have [1,31]:

(1) If p≠2, there are precisely three non-isomorphic quadratic extensions of $Q_{p}$, i.e., $Q_{p,\mu }$, with μ∈{η,p,ηp}, and where $\eta \in Q_{p}$ is a *non-quadratic unit*, i.e., $\eta \notin {\left(Q_{p}^{*}\right)}^{2}$, and ${\left|\eta \right|}_{p}=1$;
(2) if p=2, there are precisely seven non-isomorphic quadratic extensions of $Q_{p}$, i.e., $Q_{p,\mu }$, with μ∈{2,3,5,6,7,10,14}.

**Example 2.**  *For p≡3 (mod4), as a non-quadratic element in $Q_{p}$, one can take η=−1. For p≡5 (mod8), or p≡3 (mod8), η=2 is non-quadratic in $Q_{p}$ [1].*

## 3. *p*-Adic Hilbert Spaces and Operators

This section is devoted to introduce a suitable notion of a *p*-adic Hilbert space and the associated *p*-adic linear operators [31] (compare with [39,40], where different notions of non-Archimedean Hilbert spaces are introduced, and with [41], where orthogonal and symmetric operators in the non-Archimedean setting are studied).

### 3.1. p-Adic Hilbert Spaces

As is well known, complex Hilbert spaces are defined as (complex) Banach spaces endowed with a suitable inner product, namely, the one inducing the relevant norm. It turns out that this familiar picture keeps some of its main futures—but also requires some essential modification—when switching to the field of *p*-adic numbers. We start by setting the following:

**Definition 1.**  *Let $Q_{p,\mu }$ be a quadratic extension of the field of p-adic numbers $Q_{p}$. By a p-adic normed space, we mean a pair $\left(X,∥\cdot ∥\right)$, where X is a vector space over $Q_{p,\mu }$, while $∥\cdot ∥:X\to R^{+}$ is an ultrametric norm, i.e., a map satisfying the following conditions:*

(N1) $∥x∥=0$*iff*x=0*;*
(N2) $∥\alpha x∥=\left|\alpha \right|\;∥x∥$*;*
(N3) $∥x+y∥\leq \max\left\{∥x∥,∥y∥\right\}$*,*

*for all x,y∈X and $\alpha \in Q_{p,\mu }$. A p-adic normed space which is complete w.r.t. the ultrametric associated with $∥\cdot ∥$, is called a p-adic Banach space.*

**Remark 1.**  *The explicit definition of a p-adic Banach space is motivated by the fact that the strong triangle inequality *(N3)* differs significantly w.r.t. the standard (real or complex) case, where the usual triangle inequality holds.*

Let $\left(X,∥\cdot ∥\right)$ be a *p*-adic normed space. Our first concern is to provide a suitable notion of a *basis* for this space [31,34,37,42]. To this end, let us start by recalling that two vectors x,y in a *p*-adic normed space *X* are said to be (mutually) *norm-orthogonal* if, for any $\alpha ,\beta \in Q_{p,\mu }$, we have that $∥\alpha x+\beta y∥=\max\left\{∥\alpha x∥,∥\beta y∥\right\}$. Moreover, an arbitrary subset B of *X* is norm-orthogonal if any finite subset of B is such; namely, if for every set $\left\{x_{1},\ldots ,x_{n}\right\}$ of elements in B, and every set $\left\{\alpha_{1},\ldots ,\alpha_{n}\right\}$ in $Q_{p,\mu }$, we have that

(13) $$∥\sum_{i=1}^{n}\alpha_{i}x_{i}∥=\max_{1\leq i\leq n}\left|\alpha_{i}\right|\;∥x_{i}∥.$$

We say that a subset B of *X* is *normal*, if it is norm-orthogonal and, additionally, $∥x∥=1$, for all x∈B. Let now $\left(X,∥\cdot ∥\right)$ be a *p*-adic Banach space, and let $B={\left\{b_{i}\right\}}_{i\in I}\subset X\backslash \left\{0\right\}$ be a *countable* subset of *X* (i.e., we set I={1,…,N}, for some N∈N, in the case where this set is *finite*; otherwise, I=N). We say that B is a *norm-orthogonal (normal) basis*, if B is a norm-orthogonal (normal) set, and every x∈X can be expressed—in a unique way—as

(14) $$x=\sum_{i\in I}\alpha_{i}b_{i},\;\alpha_{1},\alpha_{2},\ldots \in Q_{p,\mu }.$$

In such a case, we define the *dimension* of *X*—in symbols, dim(X)—to be the (countable) cardinality of any norm-orthogonal basis in *X*, i.e., we set dim(X)=card(I). In the following, we call a *p*-adic Banach space *X* admitting a normal basis a *normal p-adic Banach space*.

**Example 3.**  *Let us consider the space $c_{0}\left(I,Q_{p,\mu }\right)$, of zero-converging sequences in $Q_{p,\mu }$:*

(15) $$c_{0}\left(I,Q_{p,\mu }\right)≔\{x={\left\{x_{i}\right\}}_{i\in I}|x_{i}\in Q_{p,\mu },\;\lim_{i}|x_{i}\left|=0\right\}.$$

*(In the case where I={1,…,N} is finite, we set $\lim_{i}\left|x_{i}\right|\equiv 0$). This set is a vector space over $Q_{p,\mu }$, and it becomes a p-adic Banach space once it is endowed with the so-called ‘sup-norm’, which is defined as*

(16) $${∥x∥}_{\infty }≔\underset{i\in I}{\sup}|x_{i}|=\max_{i\in I}\left|x_{i}\right|,\;\forall x\in c_{0}\left(I,Q_{p,\mu }\right).$$

*A normal basis for $c_{0}\left(I,Q_{p,\mu }\right)$ is given by the set $B={\left\{b_{i}\right\}}_{i\in I}$ (the so-called standard basis of $c_{0}\left(I,Q_{p,\mu }\right)$), where*

(17) $$b_{1}=\left(1,0,0,\ldots \right),\;b_{2}=\left(0,1,0\ldots \right),\;b_{3}=\left(0,0,1,\ldots \right),\;\ldots .$$

As in the standard complex case, also in the *p*-adic setting an essential step in the definition of a *p*-adic Hilbert space is the introduction of a suitable notion of *inner product*. In particular, we set the following:

**Definition 2.**  *Let $\left(X,∥\cdot ∥\right)$ be a p-adic Banach space over $Q_{p,\mu }$. By a non-Archimedean inner product we mean a map $\langle \;\cdot \;,\cdot \;\rangle :X\times X\to Q_{p,\mu }$ such that, for all x,y∈X and $\alpha ,\beta \in Q_{p,\mu }$,*

(a) *〈x,αy+βz〉=α〈x,y〉+β〈x,z〉 (linearity in the second argument);*
(b) *$\langle x,y\rangle =\bar{\langle y,x\rangle }$ (Hermitianity);*
(c) *$\left|\langle x,y\rangle \right|\leq ∥x∥∥y∥$ (Cauchy-Schwarz inequality).*

*We call the triple $\left(X,∥\cdot ∥,\langle \;\cdot \;,\cdot \;\rangle \right)$ where 〈·,·〉 is a non-Archimedean inner product, an inner-product p-adic Banach space.*

From conditions (a) and (b) of Definition 2, it is clear that the inner product 〈·,·〉 is conjugate-linear in its first argument, i.e., it is a *sesquilinear form*. Also note that, from the Hermitianity condition (b), and the sesquilinearity of 〈·,·〉, it follows that 〈0,x〉=0=〈x,0〉, for all x∈X; in particular, 〈0,0〉=0. We also say that the inner product 〈·,·〉 is *non-degenerate* if the condition 〈x,y〉=0, for all y∈X, implies that x=0.

**Example 4.**  *Let $\left(X,∥\cdot ∥\right)$ be a normal p-adic Banach space, and let $B\equiv {\left\{b_{i}\right\}}_{i\in I}$ be a normal basis in X. The canonical inner product associated with B is defined as the—non-degenerate, Hermitian—sesquilinear form*

(18) $$X\times X∋\left(x,y\right)\mapsto \langle x,y\rangle \equiv {\langle x,y\rangle }_{B}\sum_{i\in I}{\bar{x}}_{i}y_{i},$$

*where $x=\sum_{i\in I}x_{i}b_{i}$ and $y=\sum_{i\in I}y_{i}b_{i}$ are the (norm converging) expansions of the vectors x and y w.r.t. the fixed normal basis B. One can easily check that this sesquilinear product verifies all the defining conditions of a non-Archimedean inner product.*

**Remark 2.**  *The notion of non-Archimedean inner product naturally leads us to a notion of inner-product orthogonality, which is distinct from the—previously introduced—norm orthogonality. Explicitly, we say that two vectors x,y, in an inner-product p-adic Banach space X, are inner-product orthogonal (IP-orthogonal, in short) if 〈x,y〉=0.*

The notion of inner-product orthogonality, introduced in Remark 2, entails the following natural extension of the notion of normal basis:

**Definition 3.**  *Let $\left(X,∥\cdot ∥,\langle \;\cdot \;,\cdot \;\rangle \right)$ be a normal inner-product p-adic Banach space. We say that a (finite or countable) sequence of vectors $\Psi \equiv {\left\{\psi_{i}\right\}}_{i\in I}$ in X is an orthonormal basis, if *Ψ* is a normal basis in X, and its elements are mutually IP-orthogonal, namely, $\langle \psi_{i},\psi_{j}\rangle =\delta_{i,j}$, ∀i,j∈I.*

We stress that the existence of an orthonormal basis in an inner-product *p*-adic Banach space *X* is, in general, not guaranteed. On the other hand, when *X* is a normal *p*-adic Banach space—where the existence of a normal basis is assumed—it is always possible to turn any given normal basis into an orthonormal one by making a suitable choice of the inner product. Indeed, it suffices to consider the canonical inner product associated with this normal basis in *X* (recall Example 4). Therefore, we have the following natural definition of Hilbert space in the *p*-adic setting:

**Definition 4.**  *A p-adic Hilbert space is a triple $\left(H,∥\cdot ∥,\langle \;\cdot \;,\cdot \;\rangle \right)$, where $\left(H,∥\cdot ∥\right)$ is a normal p-adic Banach space, and $\langle \;\cdot \;,\cdot \;\rangle \equiv {\langle \;\cdot \;,\cdot \;\rangle }_{B}$ is the canonical inner product associated with the normal basis B in X.*

From the previous definition, it is clear that a *p*-adic Hilbert space may be thought of as a normal *p*-adic Banach space endowed with a distinguished normal basis and with the associated canonical inner product. It is then not difficult to check the following two properties of a *p*-adic Hilbert space:

(H1) Every vector x∈H can be uniquely expanded w.r.t. any orthonormal basis $\Psi \equiv {\left\{\psi_{i}\right\}}_{i\in I}$ in H, namely,
  (19) $$x=\sum_{i\in I}\langle \psi_{i},x\rangle \;\psi_{i}.$$
(H2) The *non-Archimedean Parseval identity* holds true:
  (20) $$∥x∥=\max_{i\in I}\left|\langle \psi_{i},x\rangle \right|,\;\forall x\in H.$$

**Example 5.**  *Let us consider the set $c_{0}\left(I,Q_{p,\mu }\right)$ introduced in Example 3. We have already observed that it is a normal p-adic Banach space once endowed with the sup-norm ${∥\cdot ∥}_{\infty }$ and with the standard basis (17). Then, introducing the canonical inner product in $c_{0}\left(I,Q_{p,\mu }\right)$ of Example 4, we obtain a p-adic Hilbert space. In the literature [41,43], this Hilbert space is sometimes called coordinate p-adic Hilbert space, and denoted by H(I). It plays a role analogous to the role played by $\ell^{2}\left(I\right)$ for (separable) complex Hilbert spaces. There exists an isomorphism of p-adic Banach spaces between H and H(I) (dim(H)=card(I)); see [34].*

As in the complex setting, also in the *p*-adic case one can define a convenient notion of *isomorphism of Hilbert spaces* (or unitary operator, defined as a bounded operator mapping an orthonormal basis into another) [31]. Let us briefly outline this notion. Let $\left(H,∥\cdot ∥,{\langle \;\cdot \;,\cdot \;\rangle }_{B}\right)$ be a *p*-adic Hilbert space, where ${\langle \;\cdot \;,\cdot \;\rangle }_{B}$ is the canonical inner product associated with a given normal basis B. Denote by N(H) the collection of all the normal bases in H and by N(H,B)⊂N(H) the class of all normal bases that are orthonormal w.r.t. ${\langle \;\cdot \;,\cdot \;\rangle }_{B}$. A *Hilbert space automorphism* of $\left(H,∥\cdot ∥,{\langle \;\cdot \;,\cdot \;\rangle }_{B}\right)$ is a bounded linear map transforming a basis in N(H,B) into another normal basis in the same set; equivalently, a surjective norm-isometry of H onto itself that preserves the inner product ${\langle \;\cdot \;,\cdot \;\rangle }_{B}$. This notion admits a straightforward generalization to a notion of isomorphism relating *two* Hilbert spaces over $Q_{p,\mu }$ (of the same dimension). Interestingly, if C∈N(H) is such that C∉N(H,B)—i.e., N(H,B)≠N(H,C)—then $\left(H,∥\cdot ∥,{\langle \;\cdot \;,\cdot \;\rangle }_{B}\right)$ and $\left(H,∥\cdot ∥,{\langle \;\cdot \;,\cdot \;\rangle }_{C}\right)$ are *different*, but mutually *isomorphic*, *p*-adic Hilbert spaces. The *p*-adic Hilbert spaces stemming from the same *p*-adic Banach space $\left(H,∥\cdot ∥\right)$ are in a natural one-to-one correspondence with the classes of normal bases of the type N(H,B), that form a partition of the set N(H).

**Remark 3.**  *It is worth stressing that the analogies between complex and p-adic Hilbert spaces cannot be pursued too far. Indeed, quite generally, in a p-adic Hilbert space, H, the norm does not stem directly from the inner product; i.e., in general, $∥x∥\neq \sqrt{\left|\langle x,x\rangle \right|}$. Moreover, note that a p-adic Hilbert space may contain isotropic vectors, i.e., non-zero vectors x such that 〈x,x〉=0. e.g., for p≡1 (mod4), taking into account the fact that −1 is a square in $Q_{p}$ [1], let x be a vector in the p-adic Hilbert space H (dim(H)≥2), and let $\left\{\psi_{1},\psi_{2},\ldots \right\}$ be an orthonormal basis in H. Then, setting $x=\psi_{1}+\sqrt{-1}\psi_{2}$, we have that 〈x,x〉=0.*

Hereafter, borrowing the terminology from the standard (complex) quantum mechanics, we shall call a quantum system with associated *p*-adic Hilbert space H of finite dimension N a *p*-adic quNit.

### 3.2. Linear Operators

In [31], it is demonstrated that some fundamental classes of operators used in the standard formulation of quantum mechanics—e.g., bounded and trace class operators in a complex Hilbert space—can be suitably introduced in the *p*-adic framework as well, with some non-trivial differences w.r.t. the standard complex setting.

Since our main concern is to consider applications to quantum information theory, we will actually focus our attention to linear operators acting in a *finite-dimensional p*-adic Hilbert space. In this case, we only need to consider the space L(H) of all linear operators in H, and the distinction between the various classes of operators mentioned above becomes irrelevant.

Then, let H be a finite-dimensional *p*-adic Hilbert space, with dim(H)=N, and let $\Psi \equiv {\left\{\psi_{i}\right\}}_{i=1}^{N}$ be an orthonormal basis in H. Every L∈L(H) can be represented—w.r.t. $\Psi \equiv {\left\{\psi_{i}\right\}}_{i=1}^{N}$—as a matrix operator, namely,

(21) $$L=\sum_{i=1}^{N}\sum_{j=1}^{N}\langle \psi_{i},L\psi_{j}\rangle |\psi_{i}\rangle \;\langle \psi_{j}|\equiv \sum_{i=1}^{N}\sum_{j=1}^{N}L_{ij}|\psi_{i}\rangle \;\langle \psi_{j}|,$$

where $\left(L_{ij}≔\langle \psi_{i},L\psi_{j}\rangle \right)\in M_{N}\left(Q_{p,\mu }\right)$ is the matrix associated with the operator *L* and the fixed orthonormal basis Ψ (here $M_{N}\left(Q_{p,\mu }\right)$ denotes the set of N×N matrices on $Q_{p,\mu }$). Conversely, every matrix $\left(M_{ij}\right)\in M_{N}\left(Q_{p,\mu }\right)$ defines a linear operator M∈L(H) by putting

(22) $$M≔\sum_{i=1}^{N}\sum_{j=1}^{N}M_{ij}|\psi_{i}\rangle \;\langle \psi_{j}|.$$

On the space L(H), we can define a (ultrametric) norm—namely, the *operator norm*—which is given by

(23) $$∥L∥≔\underset{0\neq \phi \in H}{\sup}\frac{∥L\phi ∥}{∥\phi ∥}=\max_{1\leq i,j\leq N}\left|\langle \psi_{i},L\psi_{j}\rangle \right|.$$

Then, by means of a standard argument (cf. Theorem 6.2.1 in [33]), it is not difficult to show that the space $\left(L(H),∥\cdot ∥\right)$ is complete w.r.t. the (ultra-)metric associated with (23); i.e., $\left(L(H),∥\cdot ∥\right)$ is a *p*-adic Banach space.

**Remark 4.**  *Let us explicitly note that, by using the Dirac notation, the operator $|\psi_{i}\rangle \;\langle \psi_{j}|$ appearing in the matrix representation of L∈L(H) should be understood as the linear operator $\langle \psi_{i},\cdot \rangle |\psi_{j}\rangle$, whose action on a generic element ϕ∈H is given by $(\langle \psi_{i},\cdot \rangle |\psi_{j}\rangle )\left(\phi \right)≔\langle \psi_{i},\phi \rangle |\psi_{j}\rangle$.*

For every L∈L(H), the *adjoint*$L^{*}$ of *L* is given by

(24) $$L^{*}=\sum_{i=1}^{N}\sum_{j=1}^{N}\bar{\langle \psi_{j},L\psi_{i}\rangle }|\psi_{i}\rangle \;\langle \psi_{j}|;$$

i.e., $L^{*}$ is the operator in L(H) with matrix coefficients given by $L_{ij}^{*}\langle \psi_{i},L^{*}\psi_{j}\rangle =\bar{\langle \psi_{j},L\psi_{i}\rangle }=\bar{L_{ji}}$. As in the standard complex setting, the adjoining operation so defined is easily seen to be an *involutive automorphism* of L(H); that is, the map $L\left(H\right)∋L\mapsto L^{*}\in L\left(H\right)$ verifies the following conditions:

(25) $${\left(\alpha A+\beta B\right)}^{*}=\bar{\alpha }A^{*}+\bar{\beta }B^{*},\;{\left(AB\right)}^{*}=B^{*}A^{*},\;{\left(A^{*}\right)}^{*}=A,\;∥A∥=∥A^{*}∥,$$

for all A,B∈L(H), $\alpha ,\beta \in Q_{p,\mu }$. Therefore, we get to the conclusion that the *p*-adic Banach space $\left(L(H),∥\cdot ∥\right)$, equipped with the adjoining operation (24), has a natural structure of a *p-adic Banach ∗-algebra*. In fact, in the next section, the set L(H) will be regarded as the Banach ∗-algebra of *physical observables* of a (finite-dimensional) *p*-adic quantum system.

**Remark 5.**  *As in the complex setting, also in the p-adic case it is possible to single out the subset $L{\left(H\right)}_{\mathrm{sa}}\subset L\left(H\right)$ of selfadjoint elements of L(H), namely, the linear operators for which the additional condition*

(26) $$L_{ij}^{*}=\bar{\langle \psi_{j},L\psi_{i}\rangle }=\langle \psi_{i},L\psi_{j}\rangle =L_{ij},$$

*is verified.*

To conclude this section, we will now argue that L(H) turns out to be a *p*-adic Hilbert space. Indeed, let us first observe that given L∈L(H), we can define its *trace*, tr(L)—w.r.t. any fixed orthonormal basis $\Psi \equiv {\left\{\psi_{i}\right\}}_{i=1}^{N}$ in H—in the usual way as

(27) $$\mathrm{tr}\left(L\right)≔\sum_{i=1}^{N}\langle \psi_{i},L\psi_{i}\rangle =\sum_{i=1}^{N}L_{ii}.$$

This definition does not depend on the choice of the orthonormal basis Ψ, and it is further possible to prove that the map $\mathrm{tr}\left(\cdot \right):L\left(H\right)\to Q_{p,\mu }$ is a *linear functional*—namely, the *trace functional*—on L(H), which satisfies the usual properties as in the standard complex case. Let us now introduce the sesquilinear form

(28) $$L\left(H\right)\times L\left(H\right)∋\left(A,B\right)\mapsto {\langle A,B\rangle }_{\;\mathrm{HS}}\;\mathrm{tr}\left(A^{*}B\right)\in Q_{p,\mu }.$$

This form is *Hermitian*, because

(29) $${\langle A,B\rangle }_{\;\mathrm{HS}}\;=\mathrm{tr}\left(A^{*}B\right)=\bar{\mathrm{tr}(B^{*}A)}=\bar{{\langle B,A\rangle }_{\;\mathrm{HS}}\;}.$$

We call this Hermitian sesquilinear form the *p-adic Hilbert-Schmidt product*. Next, note that, for all A,B∈L(H), we have:

(30) $${|\langle A,B\rangle }_{\;\mathrm{HS}}\;|\;=|\mathrm{tr}\left(A^{*}B\right)|\;=\left|\sum_{i=1}^{N}\langle A\psi_{i},B\psi_{i}\rangle \right|\leq \max_{1\leq i\leq N}\left|\langle A\psi_{i},B\psi_{i}\rangle \right|\leq ∥A∥\;∥B∥;$$

i.e., ${\langle \;\cdot \;,\cdot \;\rangle }_{\;\mathrm{HS}}\;$ satisfies the Cauchy-Schwarz inequality. Hence, we conclude that ${\langle \;\cdot \;,\cdot \;\rangle }_{\;\mathrm{HS}}\;$ is a *non-Archimedean inner product*, and L(H), endowed with this sesquilinear form, is an *inner-product p-adic Banach space*.

Now, let $\Psi \equiv {\left\{\psi_{i}\right\}}_{i=1}^{N}$ be an orthonormal basis in H. We can introduce a family of linear operators {EΨij}i,j=1N defined by

(31) EΨij∑m=1N∑n=1NEmnΨij|ψm〉〈ψn|,whereEmnΨij=δimδjn;

namely, in the usual Dirac notation, EΨij=|ψi〉〈ψj|.

**Remark 6.**  *Let L∈L(H). It is clear that*

(32) 〈EΨij,L〉HS=tr(|ψj〉〈ψi|L)=∑k=1N〈ψk,ψj〉〈ψi,Lψk〉=Lij.

*From this fact, we deduce that the product ${\langle \;\cdot \;,\cdot \;\rangle }_{\;\mathrm{HS}}\;$ is non-degenerate, i.e.,*

(33) 〈EΨij,L〉HS=0,∀i,j=1,…,N⇒L=0.

We now prove that the set {EΨij}i,j=1N is an orthonormal basis in L(H). To this end, first note that {EΨij}i,j=1N in a normal set of vectors in L(H). In fact, consider that, for every finite subset ${\left\{\alpha_{jk}\right\}}_{j,k=1}^{N}$ in $Q_{p,\mu }$, we have:

(34) ∑j=1N∑k=1NαjkEΨjk=max1≤j,k≤N|αjk|.

Moreover, we also have that

(35) 〈EΨij,EΨrs〉HS=tr(|ψj〉〈ψi||ψr〉〈ψs|)=〈ψs,ψi〉〈ψj,ψr〉=δsiδjr;

i.e., {EΨij}i,j=1N is an IP-orthogonal set w.r.t. the Hilbert-Schmidt product. Finally, by noting that any L∈L(H) is written—w.r.t. the orthonormal basis $\Psi \equiv {\left\{\psi_{i}\right\}}_{i=1}^{N}$—as (cf. (21))

(36) L=∑i=1N∑j=1N〈ψi,Lψj〉|ψi〉〈ψj|=∑i=1N∑j=1N〈ψi,Lψj〉EΨij,

we see that {EΨij}i,j=1N is an *orthonormal basis* in L(H).

Summarizing, we have the following result:

**Theorem 1.**  *Given an N-dimensional p-adic Hilbert space H, the p-adic Banach space L(H)—endowed with the p-adic Hilbert-Schmidt product ${\langle \;\cdot \;,\cdot \;\rangle }_{\;\mathrm{HS}}\;$—becomes an inner-product p-adic Banach space. In particular, the triple $\left(L\left(H\right),∥\cdot ∥,{\langle \;\cdot \;,\cdot \;\rangle }_{\;\mathrm{HS}}\;\right)$ is a p-adic Hilbert space and, for every orthonormal basis $\Psi \equiv {\left\{\psi_{i}\right\}}_{i=1}^{N}$ in H, {EΨij}i,j=1N is an orthonormal basis in this space.*

## 4. Physical States and Observables

As is well known, the most general and abstract description of quantum mechanics is provided by the so-called *algebraic formulation*. This formulation essentially relies on two fundamental assumptions; namely, that every quantum system can be described by means of two main classes of objects—i.e., *states* and *observables*—mutually related by means of a natural *pairing* map. Specifically, the observables can be identified with the selfadjoint elements of an abstract non-commutative unital $C^{*}$-algebra, whereas the states are normalized positive functionals on the $C^{*}$-algebra. In particular, in the case of ordinary quantum mechanics, the $C^{*}$-algebra of observables is realized by the non-commutative unital $C^{*}$-algebra of bounded operators B(K) in a complex Hilbert space K. The associated states are realized by trace-one positive trace class operators, the so-called *density* or *statistical* operators [44,45,46,47,48,49].

Following the same route, it has been recently argued that, in *p*-adic quantum mechanics, physical states should be defined as (normalized) *involution-preserving* bounded functionals on the unital Banach ∗-algebra B(H) of bounded operators [31]. It has been further shown that the role played by the density operators in the complex case is played, in the *p*-adic setting, by the class of the so-called *p-adic statistical operators*, defined as a suitable subclass of the selfadjoint trace class operators in a *p*-adic Hilbert space H [31]. The properties, as well as the conditions, that must be satisfied by these states are ultimately ruled by a suitable model of *p*-adic probability theory [6,7,50,51].

**Remark 7.**  *Since p-adic probability theory is a rather non standard topic, for reader’s convenience, we now briefly sketch its main features. Let us first observe that both p-adic and classical probability theory arise in a natural way from a common conceptual background. In fact, in both theories, one starts by considering the set $O_{Q}=\left\{q\in Q|0\leq q\leq 1\right\}\subset Q$ of the (relative) frequencies of experimental outcomes. Then, the set where all experimental statistical distributions take their values should coincide with the closure $\mathrm{cl}\;(O_{Q})$ of $O_{Q}$, where the closure is relative to some suitable topology. In classical probability theory, one assumes that this topology is the one induced by the standard valuation on Q, so obtaining $\mathrm{cl}\;\left(O_{Q}\right)=\left[0,1\right]\subset R$. In the p-adic case, instead, we should consider the topology induced by the p-adic valuation, which now yields $\mathrm{cl}\;\left(O_{Q}\right)=Q_{p}$. This means that, all possible normalized (i.e., summing up to 1) sequences in $Q_{p}$ provide legitimate (discrete) p-adic probability distributions [6,7,50,51]. The consequences of this fact are noteworthy. Just to mention the most relevant ones, we observe that p-adic probability theory naturally involves affine—rather than convex—structures. Moreover, certain (say, rational) values of p-adic probability that, when considered in the standard real setting, would be greater than 1 or negative (therefore, inconsistent), are actually allowed in this model. e.g., the set {1,−1,−6,7} is a legitimate p-adic probability distribution, even if it is not a standard probability distribution.*

**Definition 5.**  Let H be an N-dimensional p-adic Hilbert space. By a p-adic statistical operator we mean a linear operator ρ∈L(H), such that $\rho =\rho^{*}$ and tr(ρ)=1. Equivalently, ρ is a trace-one selfadjoint linear operator in H. We denote by

(37) $$S\left(H\right)≔\{\rho \in L\left(H\right)|\rho =\rho^{*},\;\;\mathrm{tr}\left(\rho \right)=1\},$$

*the set of p-adic statistical operators in H.*

It is convenient, at this point, to better clarify the statistical interpretation of *p*-adic quantum mechanics. To begin with, we need to introduce a suitable notion of *observable* in the *p*-adic setting. In particular, as argued in [31], a convenient mathematical tool for the description of a quantum measurement in the *p*-adic setting is provided by the so-called (discrete) *selfadjoint operator valued measures* (SOVMs) in H. A SOVM may be regarded as a suitable *p*-adic counterpart of a POVM in a complex Hilbert space. In the finite-dimensional setting we are considering, a discrete SOVM can be defined as a family $M\equiv {\left\{M_{i}\right\}}_{i\in I}$—where *I* is a finite index set—of selfadjoint operators in H such that $\sum_{i\in I}M_{i}=\mathrm{Id}$.

**Remark 8.**  *We remark that, as for a discrete POVM, a discrete SOVM should actually be defined as an additive measure on the algebra of subsets of the index set I (the power set of I), by putting, e.g., for j≠k, $M_{\left\{j,k\right\}}=M_{j}+M_{k}$. The definition of SOVM on a general measurable space is beyond the aims of the present contribution.*

**Remark 9.**  *We stress that, in p-adic quantum mechanics, there is no straightforward counterpart of a POVM of standard (complex) quantum mechanics. In fact, the field of p-adic numbers $Q_{p}$ is not ordered. As a consequence, there is no natural notion of positivity in $Q_{p}$. Accordingly, there is no natural way to define positive operators in a p-adic Hilbert space.*

As a further step, we need to specify the *pairing* between states (i.e., *p*-adic statistical operators) and observables. To this end, similarly to the standard complex case, by means of the trace functional $\mathrm{tr}\left(\cdot \right):L\left(H\right)\to Q_{p,\mu }$ we can associate, with any fixed ρ∈S(H), the linear functional $\omega_{\rho }$ on L(H) defined by

(38) $$L\left(H\right)∋B\mapsto \omega_{\rho }\left(B\right)\mathrm{tr}\left(\rho B\right)\in Q_{p,\mu }.$$

Now, taking into account the defining conditions $\rho =\rho^{*}$ and tr(ρ)=1, one can easily check that the following two conditions of $\omega_{\rho }$ hold true:

(39) $$\omega_{\rho }\left(B^{*}\right)=\bar{\omega_{\rho }\left(B\right)},\;\;\forall B\in L\left(H\right),\;\omega_{\rho }\left(\mathrm{Id}\right)=1.$$

That is, for every ρ∈S(H), $\omega_{\rho }$ is a *normalized involution-preserving* linear functional on L(H). Then, we reach the following two conclusions:

- The trace functional tr(·) provides a well defined *pairing* between *p*-adic statistical operators and observables.
- For every ρ∈S(H), and every SOVM $M\equiv {\left\{M_{i}\right\}}_{i\in I}\subset L{\left(H\right)}_{\mathrm{sa}}$, relations (39) guarantee that the sequence ${\left\{\omega_{\rho }\left(M_{i}\right)=\mathrm{tr}\left(\rho M_{i}\right)\right\}}_{i\in I}$ is a *p-adic probability distribution*.

We have then obtained a complete description of the statistical content of the theory.

We next turn our attention to the characterization of a suitable *p*-adic counterpart of the complex qubit. To this end, let us first note that, since H is finite-dimensional, by considering the matrix representation—w.r.t. a fixed orthonormal basis $\Psi \equiv {\left\{\psi_{i}\right\}}_{i=1}^{N}$ in H—of any linear operator in H, it is clear that $L\left(H\right)≅M_{N}\left(Q_{p,\mu }\right)$; i.e., one can identify the set L(H) with the set $M_{N}\left(Q_{p,\mu }\right)$ of N-dimensional matrices in $Q_{p,\mu }$. In particular, the set S(H) can be identified with the following set of N×N matrices:

(40) $$S_{N}\left(Q_{p,\mu }\right)≔\left\{Q=\left(Q_{rs}\right)\in M_{N}\left(Q_{p,\mu }\right)|\mathrm{tr}\left(Q\right)=1,\;\;Q_{rs}=\bar{Q_{sr}}\right\}.$$

Namely, we identify S(H) with the set $S_{N}\left(Q_{p,\mu }\right)$ of *trace-one p-adic Hermitian N×N matrices*. Let us focus on the particular case where N=2. We can give a complete characterization of $S\left(H\right)\equiv S_{2}\left(Q_{p,\mu }\right)$ as follows. Let

(41) $$Q=\left(\begin{array}{cc} x_{1}+\sqrt{\mu }y_{1} & x_{2}+\sqrt{\mu }y_{2}\\ x_{3}+\sqrt{\mu }y_{3} & x_{4}+\sqrt{\mu }y_{4} \end{array}\right)$$

be a matrix in $M_{2}\left(Q_{p,\mu }\right)$. We first consider the most general form of a *traceless p*-adic Hermitian matrix. In particular, the conditions tr(Q)=0 and $Q=Q^{*}$ immediately yield the following relations for *Q*:

(42) $$x_{4}=-x_{1},\;x_{3}=x_{2},\;y_{3}=-y_{2},\;y_{1}=y_{4}=0.$$

From these conditions, we deduce that a two-dimensional *p*-adic Hermitian matrix, with zero trace, is given by:

(43) $$Q=\left(\begin{array}{cc} x_{1} & x_{2}+\sqrt{\mu }y_{2}\\ x_{2}-\sqrt{\mu }y_{2} & -x_{1} \end{array}\right).$$

Next, let us introduce the *p-adic Pauli matrices*$\sigma_{1},\sigma_{2},\sigma_{3}\in M_{2}\left(Q_{p,\mu }\right)$, defined by

(44) $$\sigma_{1}≔\left(\begin{array}{cc} 1 & 0\\ 0 & -1 \end{array}\right),\;\sigma_{2}≔\left(\begin{array}{cc} 0 & 1\\ 1 & 0 \end{array}\right),\;\sigma_{3}≔\left(\begin{array}{cc} 0 & \sqrt{\mu }\\ -\sqrt{\mu } & 0 \end{array}\right).$$

Exploiting these matrices, we can rewrite *Q* in a more compact form:

(45) $$Q=x_{1}\sigma_{1}+x_{2}\sigma_{2}+x_{3}\sigma_{3}.$$

(Here we have set $y_{2}\equiv x_{3}$). It is then clear that

(46) $$\rho =\frac{1}{2}\left({\mathrm{Id}}_{2}+Q\right)=\frac{1}{2}\left({\mathrm{Id}}_{2}+x_{1}\sigma_{1}+x_{2}\sigma_{2}+x_{3}\sigma_{3}\right)$$

—where ${\mathrm{Id}}_{2}$ denotes the identity matrix in $M_{2}\left(Q_{p,\mu }\right)$—gives the most general form a two-dimensional trace-one *p*-adic Hermitian matrix in $M_{2}\left(Q_{p,\mu }\right)$. Therefore, we conclude that the set of all states of a two-dimensional *p*-adic quantum systems is

(47) $$S_{2}\left(Q_{p,\mu }\right)=\left\{\rho \in M_{2}\left(Q_{p,\mu }\right)|\rho =\frac{1}{2}\left({\mathrm{Id}}_{2}+x_{1}\sigma_{1}+x_{2}\sigma_{2}+x_{3}\sigma_{3}\right),\;\;x_{1},x_{2},x_{3}\in Q_{p}\right\},$$

where $\sigma_{1},\sigma_{2},\sigma_{3}$ are the *p*-adic Pauli matrices defined in (44). In particular, we find out that a qubit state can be represented, in the *p*-adic setting, as

(48) $$\rho =\frac{1}{2}\left(\begin{array}{cc} 1+x_{1} & x_{2}+\sqrt{\mu }x_{3}\\ x_{2}-\sqrt{\mu }x_{3} & 1-x_{1} \end{array}\right).$$

From the matrix representation (48), we observe that the *p*-adic qubit shares some analogies with the qubit states of standard quantum mechanics. In particular, the matrix representation of a *p*-adic qubit is essentially the same as in the complex case, the main formal difference consisting in the presence, in the *p*-adic case, of $\sqrt{\mu }$. However, there are two *substantial* differences between the *p*-adic and the complex case.

As a first point, note that $S_{2}\left(Q_{p,\mu }\right)$ is a norm-*unbounded* subset of $M_{2}\left(Q_{p,\mu }\right)$. Moreover, let us compute the eigenvalues of the *p*-adic qubit (48). As is easily verified, they are given by

(49) $$\lambda_{\pm }=1\mp \sqrt{x_{1}^{2}+x_{2}^{2}-\mu x_{3}^{2}},$$

where one should require that $x_{1}^{2}+x_{2}^{2}-\mu x_{3}^{2}$ is a *quadratic element* of $Q_{p,\mu }$. On the other hand, it is a well known fact that the field of *p*-adic numbers (and its quadratic extensions) is not *algebraically closed*, namely, not every non-constant polynomial admits a root in $Q_{p}$. Let us clarify this point by means of an explicit example. Take p=2, and consider the quadratic extension of $Q_{2}$ by $\sqrt{2}$, i.e., the field $Q_{2,2}$ (see Section 2). Now, consider the qubit state associated with the parameters $\left(x_{1},x_{2},x_{3}\right)=\left(4,4,3\right)$. Then, from its characteristic polynomial, we obtain the following two *formal* eigenvalues

(50) $$\lambda_{\pm }=1\mp \sqrt{x_{1}^{2}+x_{2}^{2}-\mu x_{3}^{2}}=1\mp \sqrt{14}=1\mp \sqrt{7}\sqrt{2}.$$

However, 7 in not a quadratic element of $Q_{2}$; that is, the characteristic polynomial of the matrix (48) *does not admit any root* in the quadratic extension of $Q_{2}$ just considered. Otherwise stated, we have constructed an example of a 2-adic qubit state that is *not diagonalizable*. Actually, it is not difficult to construct examples of non-diagonalizable qubit states also for all other quadratic extensions of $Q_{2}$ (as classified in Section 2). The same fact holds true also for p>2. Namely, for suitable values of $x_{1},x_{2},x_{3}\in Q_{p}$, it is possible to construct *p*-adic qubit states that cannot be diagonalized, for every quadratic extension $Q_{p,\mu }$ of $Q_{p}$.

**Example 6.**  *We now provide an explicit example of a SOVM for a two-dimensional p-adic quantum system. Let us consider the family of linear (matrix) operators $M=\{M_{1},M_{2},M_{3},M_{4},M_{5}\}$, where*

(51) $$\begin{array}{cc} \; & M_{1}≔\left(\begin{array}{ccc} 1 & \; & 0\\ 0 & \; & 1 \end{array}\right),\;M_{2}≔\left(\begin{array}{ccc} -1 & \; & 0\\ 0 & \; & 1 \end{array}\right),\;M_{3}≔\left(\begin{array}{ccc} 0 & \; & -1\\ -1 & \; & 0 \end{array}\right),\\ \; & M_{4}≔\left(\begin{array}{ccc} 0 & \; & -\sqrt{\mu }\\ \sqrt{\mu } & \; & 0 \end{array}\right),\;M_{5}≔\left(\begin{array}{ccc} 1 & \; & 1+\sqrt{\mu }\\ 1-\sqrt{\mu } & \; & -1 \end{array}\right). \end{array}$$

*It is clear that the matrices $M_{i}$, i=1,…,5, are Hermitian. Moreover,*

(52) $$\sum_{i=1}^{5}M_{i}=M_{1}+M_{2}+M_{3}+M_{4}+M_{5}=\left(\begin{array}{ccc} 1 & \; & 0\\ 0 & \; & 1 \end{array}\right)={\mathrm{Id}}_{2}.$$

*Therefore, M is indeed a SOVM. The statistical output of a measurement associated with M, when the physical system is in the p-adic qubit state (48), is*

(53) $${\left\{\mathrm{tr}\left(\rho M_{i}\right)\right\}}_{i=1}^{5}=\left\{1,\;-x_{1},\;-x_{2},\;\mu x_{3},\;x_{1}+x_{2}-\mu x_{3}\right\},$$

*with $x_{1},x_{2},x_{3}\in Q_{p}$. Since $\sum_{i=1}^{5}\mathrm{tr}\left(\rho M_{i}\right)=1$, we have that $\left\{1,\;-x_{1},\;-x_{2},\;\mu x_{3},\;x_{1}+x_{2}-\mu x_{3}\right\}$ is a p-adic probability distribution.*

## 5. Conclusions

As a first step towards a quantum information theory based on a quadratic extension of the non-Archimedean field of *p*-adic numbers, we have proposed a model of QuNit on the field $Q_{p,\mu }$, where μ is a non-square element of $Q_{p}$.

We started by introducing a notion of *p*-adic Hilbert space and, restricting to the case where H is finite-dimensional, the associated space of linear operators L(H). Then, we have described various properties of the ultrametric Banach space L(H). We have argued that L(H), endowed with the operator norm and the adjoining operation, turns out to be a *p-adic Banach ∗-algebra*. Then, we have proved that the linear space L(H) itself has a natural structure of a *p*-adic Hilbert space, once it is endowed with the *p-adic Hilbert-Schmidt inner product*.

Owing to the distinguishing features of *p*-adic probability theory, we have argued that the states of an N-dimensional *p*-adic quantum system are implemented by *p-adic statistical operators*, i.e., trace-one selfadjoint operators in the carrier Hilbert space. In particular, it turns out that the set of *p*-adic statistical operators, S(H), is a *$Q_{p}$-affine* subset of L(H)—coherently with the affine structure of *p*-adic probability theory—hence, it is an *unbounded* subset of L(H).

We have next introduced the notion of (discrete) selfadjoint-operator-valued measure (SOVM)—a suitable *p*-adic counterpart of a POVM in a complex Hilbert space—as a convenient mathematical tool describing the physical observables of a *p*-adic quantum system.

Eventually, focusing on the special case where N=2, we have provided a description of *p*-adic qubit states and of two-dimensional SOVMs.

We close by outlining some potential extensions of this work, especially focusing on those ones that are relevant for our (final) program aimed at developing a *p*-adic model of quantum information theory. Tensor products and entanglement play a central role in quantum information theory, and we expect that they will play a central role in the *p*-adic setting too. Therefore, as a first step, we plan to investigate tensor products of *p*-adic Hilbert spaces and the associated classes of *entangled states*. Our next concern is the description of dynamical maps and dynamical (semi-)groups in *p*-adic quantum mechanics. This will provide a suitable *p*-adic counterpart to quantum channels. Another interesting prospect concerns the possibility of defining typical entropic quantities, such as the von Neumann and the Rényi entropies—which are relevant in standard quantum information theory—in the *p*-adic framework too.

## Data Availability

Not applicable.
